# *atpE* Mutation in *Mycobacterium tuberculosis* Not Always Predictive of Bedaquiline Treatment Failure

**DOI:** 10.3201/eid2805.212517

**Published:** 2022-05

**Authors:** Laure Fournier Le Ray, Alexandra Aubry, Wladimir Sougakoff, Matthieu Revest, Jérôme Robert, Isabelle Bonnet, Nicolas Veziris, Florence Morel

**Affiliations:** Sorbonne Université, Institut National de la Santé et de la Recherche Médicale, Paris, France (L. Fournier Le Ray, A. Aubry, W. Sougakoff, J. Robert, I. Bonnet, N. Veziris, F. Morel);; Hôpital Pitié-Salpêtrière, Assistance Publique–Hôpitaux de Paris, Sorbonne Université, Paris, France (A. Aubry, W. Sougakoff, J. Robert, I. Bonnet, F. Morel);; Pontchaillou University Hospital, Rennes, France (M. Revest);; Université de Rennes, Institut National de la Santé et de la Recherche Médicale, Rennes (M. Revest);; Hôpital Saint-Antoine, Assistance Publique–Hôpitaux de Paris, Sorbonne Université, Paris (N. Veziris)

**Keywords:** tuberculosis, atpE, bedaquiline, MDR TB, XDR TB, Rv0678, antimicrobial resistance, bacteria, tuberculosis and other mycobacteria

## Abstract

We report the emergence of an *atpE* mutation in a clinical *Mycobacterium tuberculosis* strain. Genotypic and phenotypic bedaquiline susceptibility testing displayed variable results over time and ultimately were not predictive of treatment outcome. This observation highlights the limits of current genotypic and phenotypic methods for detection of bedaquiline resistance.

Bedaquiline is one of the core drugs used to treat multidrug-resistant (MDR) tuberculosis (TB) and extensively drug-resistant TB (XDR TB) (*1*). Bedaquiline resistance is now part of the revised definition of XDR TB, and its incidence is rising alarmingly (*2*,*3*). Resistance to bedaquiline is mainly caused by mutations in *Rv0678* (*mmpR*), which encodes the repressor of the efflux pump MmpL5−MmpS5, usually leading to low-level resistance (*4*). Conversely, mutations in *atpE*, which encodes the target of bedaquiline, the c subunit of the ATP synthase, are rarely described in clinical strains (*5*) and are associated with high increase of MICs (*4*). Mutations in *pepQ* and *Rv1979c* are also reported, but their effect on bedaquiline susceptibility is unclear. We report a case of an *atpE* mutation in a bedaquiline-resistant clinical strain of *Mybobacterium tuberculosis* and discuss the performances of current methods for susceptibility testing (Appendix) and their clinical implications (*6*). 

A 32-year-old man from Georgia received a diagnosis of bilateral cavitary lung MDR TB upon his arrival in France in January 2020. Three consecutive treatment regimens of bedaquiline and clofazimine had failed. A fourth regimen combining bedaquiline, linezolid, cycloserine, clofazimine, delamanid, and amoxicillin/clavulanate + meropenem was initiated on arrival.

The first isolate from January 2020 (S1) was bedaquiline resistant with a MIC dilution above the breakpoint (MIC 2 mg/L) and clofazimine-susceptible with a MIC close to the breakpoint (MIC 1 mg/L). We detected 2 deletions (P129fs [15%] and G66fs [54%]) in *Rv0678 *(Figure).

**Figure:**
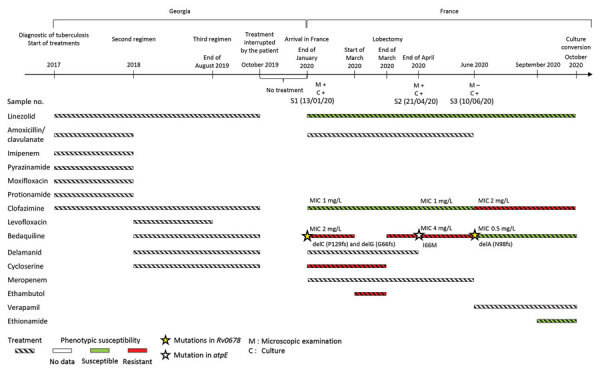
Timeline of treatment regimen and microbiologic data for patient with multidrug-resistant tuberculosis before and after his arrival in France from Georgia. Timeline for each antibiotic indicates treatment (striped), phenotypic resistance (red), and susceptibility (green), as indicated in patient records. M, microscopic examination; C, culture.

At the end of March 2020, cycloserine was withdrawn from the patient’s treatment regimen because of phenotypic resistance, and bedaquiline, which had been stopped 1 month earlier, was resumed; the patient underwent a lobectomy. One month after the procedure, sputum microscopic examination and culture were still positive. The second isolate (S2) from April 2020 had an increased bedaquiline MIC (4 mg/L), but clofazimine MIC remained unchanged (1 mg/L). No mutation in *Rv0678* was detected, but we observed an AtpE I66M (63%) substitution.

Two months later in June, the patient was sputum smear negative but remained culture positive. Isolate S3 was susceptible to bedaquiline (MIC 0.5 mg/L) and clofazimine resistant (MIC 2 mg/L). A deletion was found in *Rv0678* different from those identified in S1: deletion at position 293 (N98fs) (97%), whereas no mutation was identified in *atpE*. Verapamil and ethionamide were added and amoxicillin/clavulanate + meropenem stopped. Finally, samples from September 2020 were culture negative, with regression of pulmonary lesions. The outcome was classified as treatment success in February 2021 after 13 months of treatment and was still favorable as of December 2021.

All 3 isolates shared the same spoligotype (SIT1) (Beijing lineage) and displayed only 3 single-nucleotide variants (SNVs) of difference by pairwise comparison. The SNVs were all nonsynonymous. Two SNVs were only recovered in strain S2, 1 corresponding to the AtpE: I66M substitution and 1 located in *Rv0243* (L136P substitution) encoding the acetyl−CoA acyltransferase FadA2 and probably implicated in lipid degradation. One SNV was only found in S1 in *Rv3909* (M683L substitution), encoding a protein of unknown function. No mutations were observed in *pepQ*, its promoter, or in *Rv1979c* (*7*).

As this case illustrates, identifying bedaquiline resistance in the laboratory and its effects on patient management appear complex. Over a 6-month period, we tested 3 *M. tuberculosis* isolates with different genotypic and phenotypic patterns regarding bedaquiline, exhibiting various MIC levels and mutations in genes involved in bedaquiline resistance. These isolates displayed only 3 SNVs by pairwise comparison of their genomes, excluding a reinfection by a new strain or a mixed infection.

Of note, mutations in *atpE* or *Rv0678* were found only once and were not found at subsequent timepoints. Despite continuous bedaquiline treatment, resistant strain S2 with the *atpE* mutation was not selected, and the patient was cured. A previous in vitro study suggested that, whereas *Rv0678* mutations were dynamic over time, *atpE* mutations were fixed once they appeared (*8*). This observation was not confirmed by our clinical case. One possible explanation for nonfixation of these mutations could be the associated fitness cost. However, an in vitro study did not show any fitness cost because of the I66M substitution (*9*). Because fitness also depends on the genetic background, the results of this in vitro study might not be transposable here. Regarding *Rv0678*, 2 mutations have been studied and did not have fitness impact (E138G and R94Q) (*4*). Additional in vivo and epidemiologic studies would help evaluate the fitness cost of such mutations. Another explanation for the variability of genotypic and phenotypic bedaquiline susceptibility over time could be a spatial heterogeneity in the lesions as already described (*10*).

This case raises concerns about the ability of current phenotypic and genotypic methods to detect bedaquiline resistance. Further studies are needed before relying on these methods for therapeutic decisions. In the meantime, these data can help improve the World Health Organization database of drug resistance–related mutations (*11*). Overall, this case underlines the complexity of bedaquiline-resistance mechanisms and of the dynamics of mutation emergence and selection.

Appendix Additional information about *atpE* mutation in *Mycobacterium tuberculosis* as unreliable predictor of bedaquiline treatment failure

## References

[1] Van Deun A, Decroo T, Piubello A, de Jong BC, Lynen L, Rieder HL. Principles for constructing a tuberculosis treatment regimen: the role and definition of core and companion drugs. Int J Tuberc Lung Dis. 2018;22:239–45. 10.5588/ijtld.17.0660 29471899

[2] World Health Organization. Meeting report of the WHO expert consultation on the definition of extensively drug-resistant tuberculosis, 27–29 October 2020 [cited 2021 Feb 5]. https://www.who.int/publications/i/item/meeting-report-of-the-who-expert-consultation-on-the-definition-of-extensively-drug-resistant-tuberculosis

[3] Andres S, Merker M, Heyckendorf J, Kalsdorf B, Rumetshofer R, Indra A, et al. Bedaquiline-resistant tuberculosis: dark clouds on the horizon. Am J Respir Crit Care Med. 2020;201:1564–8. 10.1164/rccm.201909-1819LE 32053752

[4] Andries K, Villellas C, Coeck N, Thys K, Gevers T, Vranckx L, et al. Acquired resistance of *Mycobacterium tuberculosis* to bedaquiline. PLoS One. 2014;9:e102135. 10.1371/journal.pone.0102135 25010492 PMC4092087

[5] Peretokina IV, Krylova LY, Antonova OV, Kholina MS, Kulagina EV, Nosova EY, et al. Reduced susceptibility and resistance to bedaquiline in clinical *M. tuberculosis* isolates. J Infect. 2020;80:527–35. 10.1016/j.jinf.2020.01.007 31981638

[6] World Health Organization. Technical manual for drug susceptibility testing of medicines used in the treatment of tuberculosis. 2018 [cited 2018 Oct 18]. https://apps.who.int/iris/handle/10665/275469

[7] Battaglia S, Spitaleri A, Cabibbe AM, Meehan CJ, Utpatel C, Ismail N, et al. Characterization of genomic variants associated with resistance to bedaquiline and delamanid in naive mycobacterium tuberculosis clinical strains. J Clin Microbiol. 2020;58:1304–24. 10.1128/JCM.01304-20 32907992 PMC7587096

[8] Ismail N, Ismail NA, Omar SV, Peters RPH. In vitro study of stepwise acquisition of rv0678 and atpE mutations conferring bedaquiline resistance. Antimicrob Agents Chemother. 2019;63:e00292–19. 10.1128/AAC.00292-19 31138569 PMC6658778

[9] Huitric E, Verhasselt P, Koul A, Andries K, Hoffner S, Andersson DI. Rates and mechanisms of resistance development in *Mycobacterium tuberculosis* to a novel diarylquinoline ATP synthase inhibitor. Antimicrob Agents Chemother. 2010;54:1022–8. 10.1128/AAC.01611-09 20038615 PMC2825986

[10] Kaplan G, Post FA, Moreira AL, Wainwright H, Kreiswirth BN, Tanverdi M, et al. *Mycobacterium tuberculosis* growth at the cavity surface: a microenvironment with failed immunity. Infect Immun. 2003;71:7099–108. 10.1128/IAI.71.12.7099-7108.2003 14638800 PMC308931

[11] World Health Organization. Catalogue of mutations in *Mycobacterium tuberculosis* complex and their association with drug resistance [cited 2022 Jan 18]. https://www.who.int/publications-detail-redirect/9789240028173

