# Supplementary material for: atpE Mutation in Mycobacterium tuberculosis Not Always Predictive of Bedaquiline Treatment Failure
Source: Emerg Infect Dis. 2022 May;28(5):1062–4. doi: 10.3201/eid2805.212517 (PMC9045433; doi:10.3201/eid2805.212517)
Supplement: Appendix — Additional information about atpE mutation in Mycobacterium tuberculosis as unreliable predictor of bedaquiline treatment failure [file 21-2517-Techapp-s1.pdf]

Article DOI: https://doi.org/10.3201/eid2805.212517

atpE Mutation in *Mycobacterium tuberculosis* Not Always Predictive of Bedaquiline Treatment Failure

Appendix

Phenotypic and Genotypic Methods Used for Bedaquiline and Clofazimine Susceptibility Testing

Phenotypic drug susceptibility tests were performed by the proportion method and MICs of bedaquiline and clofazimine were determined by using the TB ExiST BD BACTEC MGIT 960 System. The clinical categorization into susceptibility or resistance was based on the proposed breakpoint of 1 mg/L for both antibiotics. Genotypic DST was done by using the Deeplex Myc-TB (Genoscreen, Lille, France) and whole-genome sequencing (WGS). After genomic DNA extraction by Genelead VIII (Diagenode), the Illumina WGS was performed at the Genoscreen platform (Lille, France) (raw data available under SRA accession number PRJNA768393). Variant calling was performed on BioNumerics-7 after mapping to the H37Rv reference genome (GenBank accession AL123456.3). Single-nucleotide variations in repetitive regions such as PE_PGRS/PPE were excluded from further analysis.
